# Bring the pain: wounding reveals a transition from cortical excitability to epithelial excitability in *Xenopus* embryos

**DOI:** 10.3389/fcell.2023.1295569

**Published:** 2024-02-22

**Authors:** Leslie A. Sepaniac, Nicholas R. Davenport, William M. Bement

**Affiliations:** ^1^ Center for Quantitative Cell Imaging, University of Wisconsin-Madison, Madison, WI, United States; ^2^ Department of Integrative Biology, University of Wisconsin-Madison, Madison, WI, United States; ^3^ Cellular and Molecular Biology Graduate Program, University of Wisconsin-Madison, Madison, WI, United States

**Keywords:** cortical excitability, *Xenopus* embryo, wound healing, F-actin waves, embryonic excitability

## Abstract

The cell cortex plays many critical roles, including interpreting and responding to internal and external signals. One behavior which supports a cell’s ability to respond to both internal and externally-derived signaling is cortical excitability, wherein coupled positive and negative feedback loops generate waves of actin polymerization and depolymerization at the cortex. Cortical excitability is a highly conserved behavior, having been demonstrated in many cell types and organisms. One system well-suited to studying cortical excitability is *Xenopus laevis*, in which cortical excitability is easily monitored for many hours after fertilization. Indeed, recent investigations using *X. laevis* have furthered our understanding of the circuitry underlying cortical excitability and how it contributes to cytokinesis. Here, we describe the impact of wounding, which represents both a chemical and a physical signal, on cortical excitability. In early embryos (zygotes to early blastulae), we find that wounding results in a transient cessation (“freezing”) of wave propagation followed by transport of frozen waves toward the wound site. We also find that wounding near cell-cell junctions results in the formation of an F-actin (actin filament)-based structure that pulls the junction toward the wound; at least part of this structure is based on frozen waves. In later embryos (late blastulae to gastrulae), we find that cortical excitability diminishes and is progressively replaced by epithelial excitability, a process in which wounded cells communicate with other cells via wave-like increases of calcium and apical F-actin. While the F-actin waves closely follow the calcium waves in space and time, under some conditions the actin wave can be uncoupled from the calcium wave, suggesting that they may be independently regulated by a common upstream signal. We conclude that as cortical excitability disappears from the level of the individual cell within the embryo, it is replaced by excitability at the level of the embryonic epithelium itself.

## Introduction

The cell cortex–the plasma membrane and the cytoplasm just beneath it (Just, 1939)–receives many internal and external stimuli to which it must flexibly respond. Frequently, these responses require alterations of the cytoskeleton, such as those needed for cell protrusion, or for cytokinesis or morphogenesis. One mechanism that cells harness to direct such alterations is cortical excitability. Cortical excitability refers to the ability of the cell cortex to behave as an excitable medium such that it generates propagating waves of actin assembly and disassembly as a result of positive feedback coupled to delayed negative feedback (Vicker, 2000; Michaud et al., 2021). Such waves are often under the control of complementary waves of Rho GTPase activation and inactivation (Bement et al., 2015) that are themselves subject to complex feedback interactions (Bement et al., 2024). In recent years, improvements in imaging approaches have revealed the participation of cortical excitability in a diverse array of processes including polarized cell migration in cultured myeloid cells (Weiner et al., 2007), spindle positioning in mast cells (Xiao et al., 2017), cytokinesis in frog and starfish embryos (Bement et al., 2015), *C. elegans* polarization (Michaux et al., 2018), embryonic compaction supporting morphogenesis in the mouse embryo (Maitre et al., 2015), adhesion and spreading of U2OS cells (Graessl et al., 2017), and activation of immune cells (Colin-York et al., 2019).

Cortical excitability in embryos is of particular interest for several reasons. First, it can be unusually well-developed and constitutive. In *Xenopus* embryos, for example, high amplitude cortical waves of F-actin assembly are associated with low amplitude waves of Rho activity throughout the cell and throughout the cell cycle (Bement et al., 2015; Swider et al., 2022). At anaphase, high amplitude Rho waves form at the cell equator as a result of the concentration of the Rho GEF, Ect2, and the Rho GAP, RGA-3/4 (Bement et al., 2015; Michaud et al., 2022), where they direct formation of the cytokinetic apparatus. Meanwhile, outside the equator (i.e., in nonfurrow regions) the high amplitude actin waves and associated low amplitude Rho waves persist. And while reproducible changes in nonfurrow wave amplitude and period are keyed to and dependent on cell cycle transitions (Swider et al., 2022), these are relatively modest. Second, because embryos undergo cleavage (i.e., a halving of cell volume during each cell division cycle) scaling relationships between cortical wave features and cell size (Xiao et al., 2017) can be studied under natural conditions (Swider et al., 2022). Third, because embryos can develop over the course of hours or days prior to nervous system formation, other means of long-range communication must presumably be used to transmit or receive information. An additional reason to study cortical excitability in embryos stems from the fact that they (and their developmental precursors–oocytes and eggs) have a well-characterized cortical cytoskeletal response to wounding that involves many of the same participants employed during cortical excitability, including F-actin, myosin-2, and the small GTPases–Rho, Rac, and Cdc42 (Bement et al., 1999; Mandato and Bement, 2001; Benink and Bement, 2005; Clark et al., 2009; Abreu-Blanco et al., 2011; Burkel et al., 2012; Soto et al., 2013; Abreu-Blanco et al., 2014; Verboon and Parkhurst, 2015; Slater et al., 2021). Because the repair response includes calcium- and Rho GTPase-dependent formation and closure of a contractile array comprising F-actin and myosin-2, along with the associated flow of cortical material toward the wound (Bement et al., 1999; Mandato and Bement, 2001; Benink and Bement, 2005; Abreu-Blanco et al., 2011; Abreu-Blanco et al., 2014; Verboon and Parkhurst, 2015), wounding represents both a chemical and physical stimulus.

Previous studies of embryo repair were performed prior to the discovery of cortical excitability and at spatial and temporal resolutions insufficient to resolve traveling cortical actin waves. Accordingly, in this study we have reinvestigated the wound response in *Xenopus* embryos using an imaging regime that permits visualization of cortical excitability. We find that in early embryos, wounding elicits transient immobilization of waves around the wound site, followed by flow of waves toward the wound. We also find that wounds made near cell-cell junctions elicit formation of an F-actin based contractile structure that pulls junctions and wounds together. At later time points in embryogenesis, as cell size decreases and the natural cortical excitability dampens, the contraction and flow are replaced by a response in which wounding in one cell elicits a wave-like burst of actin assembly in neighboring cells. This response apparently results from a wave-like increase in calcium that likewise travels from the wounded cell to its neighbors. The multicellular waves elicited by wounding later stage embryos have the hallmarks of excitability, indicating that during *Xenopus* development, cortical excitability is progressively replaced by a tissue-based excitable network that allows long-range transmission of information from the site of tissue damage to distal regions of the embryo.

## Results

### Cortical excitability in early *Xenopus* embryos

**FIGURE 1:**
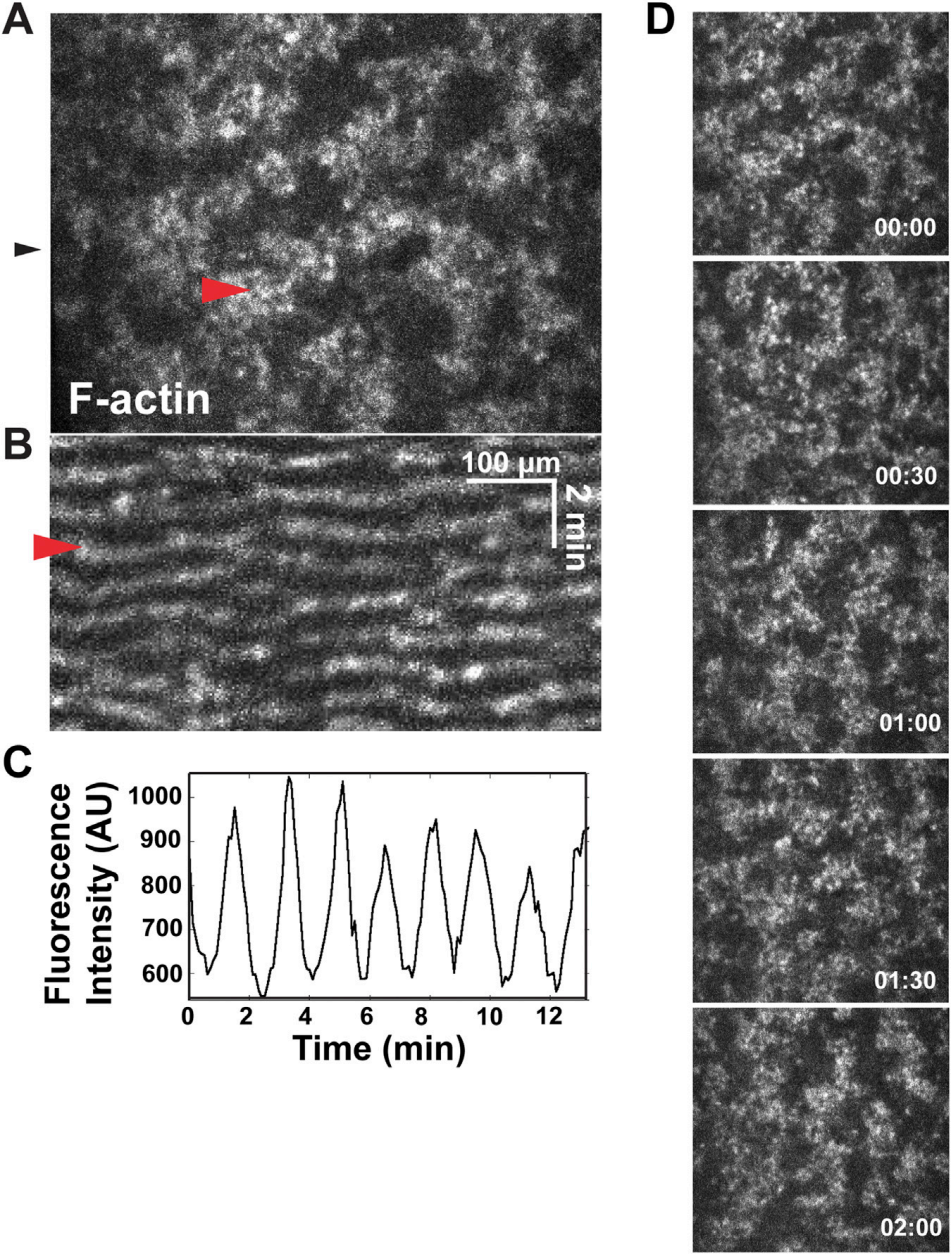
Waves of F-actin in the *Xenopus* embryo cortex. **(A)** Single frame from a movie showing confocal F-actin waves in a *Xenopus* embryo. Red arrowhead indicates single wave. **(B)** Kymograph made form the movie in A, generated from the position indicated by black arrowhead in A. Red arrowhead indicates wave. **(C)** Plot of fluorescence intensity (in arbitrary units, AU) over time (in minutes) of 6 μm^2^ of cortex. **(D)** Montage of propagating waves of F-actin across a cortex, within a representative wave period.

Cortical excitability can be easily visualized in the *Xenopus* system by imaging F-actin dynamics in either artificially activated eggs or fertilized eggs (Figures 1A–D; Bement et al., 2015; Swider et al., 2022). As shown in Figure 1 and Movie 1, cortical excitability is revealed by mobile, irregularly sized cortical patches of F-actin that translocate through the cortex. In kymographs (Figure 1B), the F-actin waves are evident as sloping lines. Previous work demonstrated that these mobile patches are Rho dependent and propagate through the cortex as waves of actin assembly and disassembly rather than transport by myosin motors (Bement et al., 2015). When waves collide with each other, they auto-annihilate (i.e., cancel each other out), which is a signature of excitable dynamics. This behavior is evident in the kymograph as two sloping lines converging with each other (Figure 1B.; see also Bement et al., 2015).

There are two classes of F-actin waves in frog embryos–furrow and nonfurrow waves (Swider et al., 2022). As their name suggests, *furrow waves* appear during anaphase and become concentrated and amplified at the equatorial cortex by the mitotic spindle, where they direct cytokinesis (Bement et al., 2015; Swider et al., 2022). *Nonfurrow waves,* in contrast, are present throughout mitosis and are found throughout the entire cortex, except during anaphase, when they become excluded from the equatorial cortex (Swider et al., 2022). Both types of waves are associated with and dependent on waves of Rho activity, but there are differences: the amplitude of Rho in furrow waves is comparatively high; in nonfurrow waves it is comparatively low, while the opposite is true for the amplitude of the actin waves (Bement et al., 2015; Swider et al., 2022).

The furrow waves do not arise until anaphase, whereas the nonfurrow waves undergo subtle but reproducible oscillations in amplitude and period that are entrained to the cell cycle. In brief, nonfurrow wave period rises through interphase and peaks just before mitotic entry. Midway through mitosis, the wave period is reset back to early interphase levels, where period once again increases as cells exit mitosis and proceed to the next interphase. Wave amplitude peaks early in interphase, remaining steady as the cell approaches mitosis, then drops to a minimum midway through mitosis. As with wave period, wave amplitude (and cortical wave occupancy, overall) also rises as cells exit mitosis (Swider et al., 2022). The wave dynamics also change over the course of development. Specifically, wave amplitude initially increases after fertilization, but then gradually declines over the course of development such that by mid-blastula to late blastula, cortical F-actin waves became undetectable (Swider et al., 2022).

### The impact of wounding on cortical excitability in early *Xenopus* embryos

Previous work demonstrated that laser wounding of cells in 2–32 cell stage embryos elicits a response similar to that seen in immature oocytes (Clark et al., 2009): Rho and Cdc42 are rapidly activated around wound sites and direct formation of a contractile ring of F-actin and myosin-2 that closes around the wound (Benink and Bement, 2005). However, as noted in the Introduction, these studies were performed prior to the discovery of cortical excitability and without sufficient spatiotemporal resolution to monitor cortical excitability. We therefore repeated wounding experiments of activated eggs and embryos using high spatiotemporal resolution time-lapse confocal microscopy as above.

**FIGURE 2:**
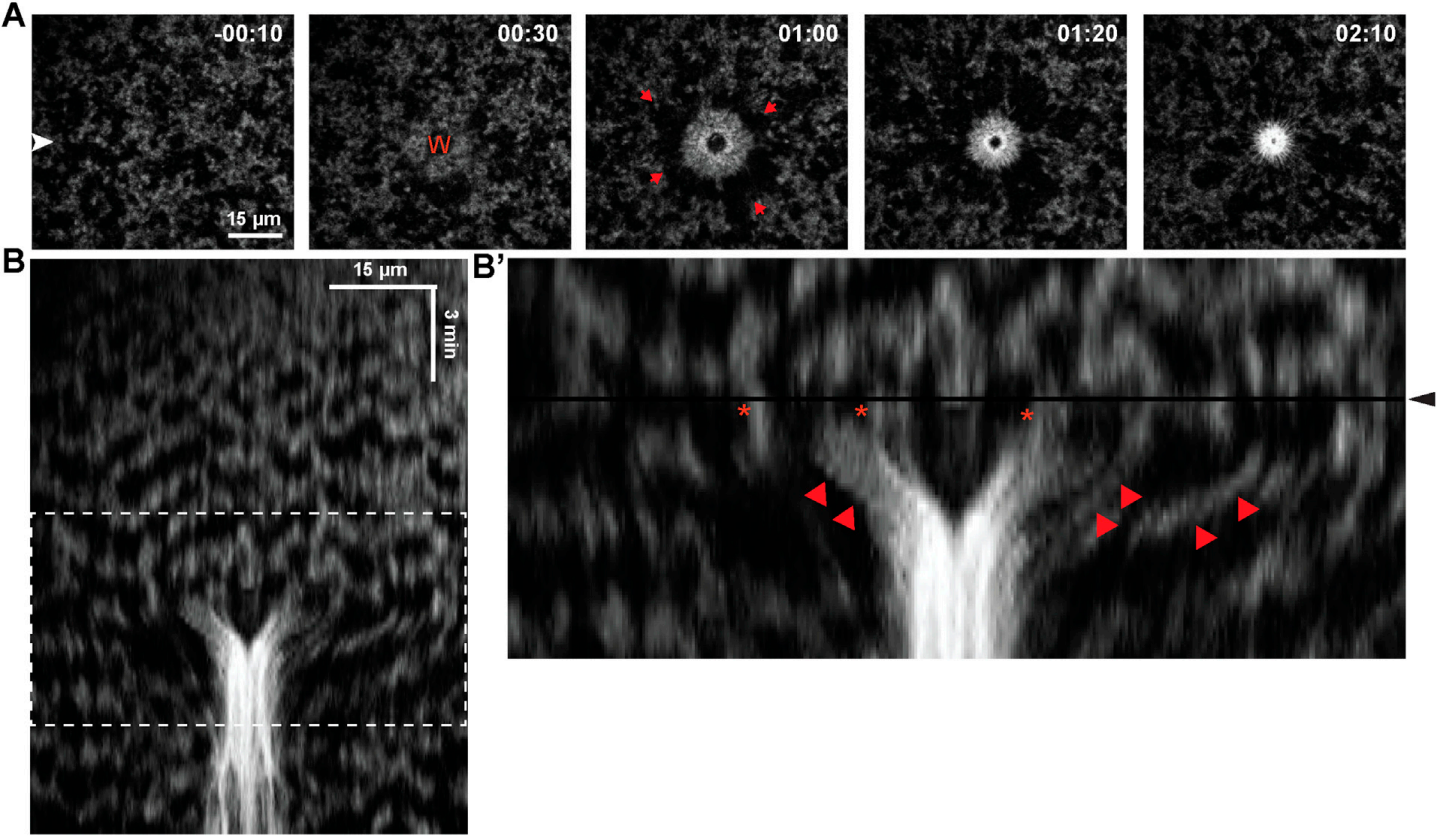
Impact of blastomere wounding on cortical F-actin waves. **(A)** Montage of blastomere surface, before and after wounding, with time (in minutes) indicated relative to wound initiation. Wound position labeled as “W”, and red arrows indicates direction of cortical flow to facilitate closure of the wound. **(B)** Kymograph of blastomere wounding. **(B′)** Enlarged portion of kymograph from **(B)**, (enlarged region indicated via dashed white box in **(B)**), where wound initiation occurs at the black horizontal line and labeled via black arrowhead. Proximal regions of F-actin waves (indicated by star) are pulled inward, to the center of the wound (indicated by red arrowhead).

Consistent with previous results (Clark et al., 2009), wounding of cells in 2–32 cell embryos elicited rapid formation of an F-actin-rich contractile ring around wounds. The ring closed inward, accompanied by cortical flow toward the wound (Figure 2A). Wounding had at least three effects on waves near the wound site: first, the waves appeared to freeze (i.e., stop propagating) immediately after wounding (Figure 2B). Second, the frozen waves were then swept toward the wound, apparently by cortical flow (Figure 2B’). Third, the region around the wound subject to cortical flow did not appear to support new wave formation which, combined with the flow, temporarily stripped the wound-proximal cortex of waves.

### The impact of wounding on cortical excitability in 32–512 cell embryos

We next assessed the consequences of wounding in later embryos (32–512 cells), at which point cell-cell junctions are better-developed. Previous work revealed that wounding at this stage could elicit a “hybrid” wound response in which wound-induced contractile rings encompass both the wounded cell and the junctions of nearby neighbor cells (Clark et al., 2009). This response depended on the distance between the wound and the cell-cell junctions such that nearly all wounds within ∼25 μm of the junctions formed hybrid contractile rings, while none that are more than 100 μm from the junctions did so (Clark et al., 2009).

**FIGURE 3:**
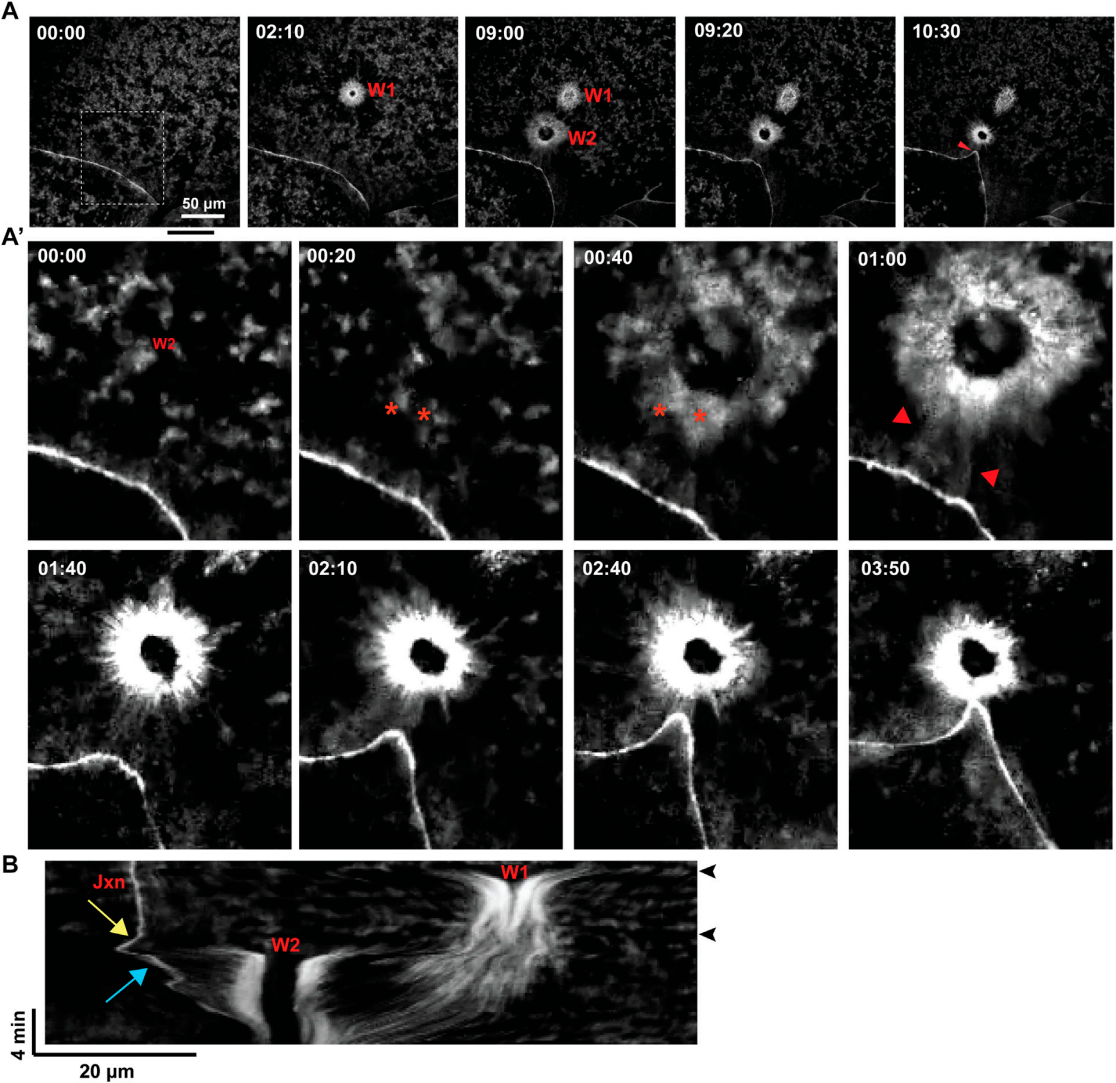
Formation of a contractile structure between wounds and junctions. **(A)** Time lapse of a late-stage embryo, highlighting a region of the cortex nearby a cell-cell junction, with time (in minutes) reported relative to the induction of the first wound. Two wounds are introduced: Wound 1 (“W1”) appears further from the junction, while Wound 2 (“W2”) which is located closer to the junction, pulls this junction. **(A′)** Enlarged view of the cortex with Wound 2 and nearby cell junction, shown in **(A)**. F-actin waves spanning the region between the wound edge and the proximal junction are indicated via star (‘*’), and these waves of F-actin are incorporated into the contractile array, creating a localized displacement of the junction as it is pulled toward the wound edge. **(B)** Kymograph of the wounding events shown in **(A)**, where Wound 2 is shown to pull the nearby junction toward Wound 2, unlike the more distal Wound 1. **(B)** Kymograph of the wounding events shown in **(A)**, where Wound 2 is shown to pull the nearby junction toward Wound 2, unlike the more distal Wound 1. Each wound initiation is shown by black horizontal line, and labeled via black arrowhead.

When wounds were made distal to cell-cell junctions, the results were similar to what was observed in the earlier blastomere experiments above, with local freezing of waves, pulling of waves toward wounds and clearing of waves from wound proximal regions but, consistent with previous work, no apparent impact on the nearest cell junction (Figure 3A). In contrast, when wounds were made proximal to cell-cell junctions, the junction began ingressing toward the wound until it eventually merged with it, forming a hybrid contractile ring (Figure 3A). While the impact of wounding on cortical excitability appeared similar to the results above, close inspection revealed that although wave freezing occurred around the entire wound, the side facing the ingressing junction was not cleared of cortical F-actin. Rather, F-actin accumulated in an array that spanned from the wound edge to the junction, with cables of F-actin seeming to bridge the wound to the junction. The frozen waves, rather than being pulled into the wound, instead appeared to become incorporated into this array.

Thus, the impact of wounding on cortical excitability depended on the position of the wound relative to the junctions. Comparison of the behavior of the junctions in the distal vs the proximal wound suggested a potential explanation. That is, the distal wounds had no obvious effect on the behavior of the nearest junction whereas the proximal wound resulted in recoil of the junction away from the wound. This difference is most easily seen in the kymograph (Figure 3B). This recoil presumably reflects a release of tension which could be transduced into a signal that results in formation of the wound-junction F-actin array (see Discussion).

### Wounding in late blastulas or early gastrulas reveals epithelial excitability.

**FIGURE 4:**
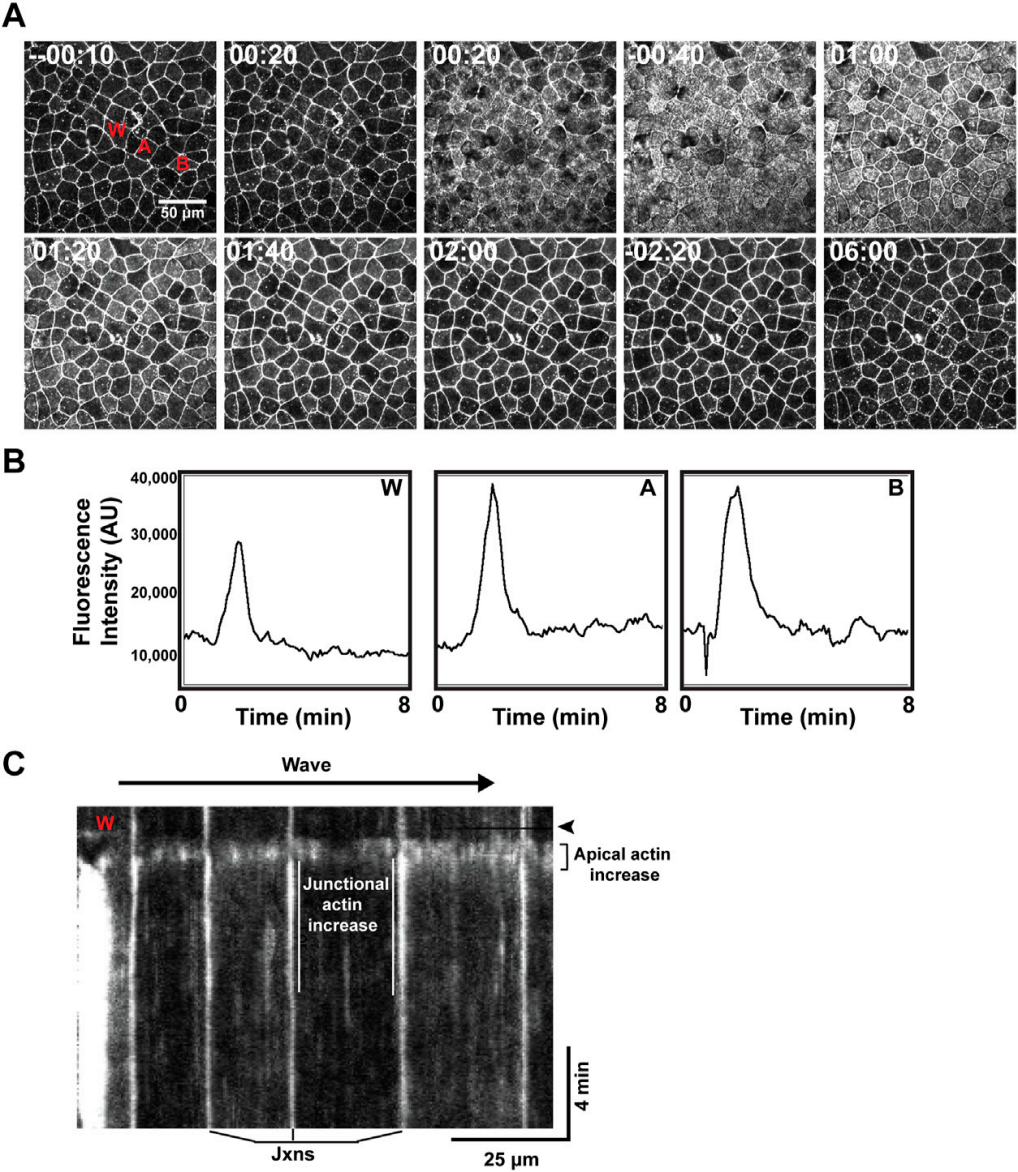
A wave of wound-induced actin assembly in gastrulae. **(A)** Time lapse imaging of the cell cortex, with time relative to wound induction, showing a wave-like response of increased apical F-actin spreading beyond the wound and remaining to reinforce nearby cell junctions. **(B)** Plot of fluorescence intensity (in arbitrary units, AU) over time (in minutes) demonstrating increased apical F-actin response at different points across the cortex (corresponding to labeling in A): “W” at the wound site; “A” indicating a cell directly neighboring the wound; “B” indicating a neighboring cell positioned further from the wound. Increased apical F-actin is shown to occur at a similar time point and magnitude across these locations. **(C)** Kymograph of wound response, for example, shown in **(A)**. Wound site labeled as “W” and timepoint of wound induction indicated by black horizontal line and emphasized via black arrowhead. Junctional actin increase is emphasized via white parallel lines, framing the increase in F-actin signal occurring at the junctions (labeled below). A “flash” of apical F-actin is highlighted, occurring almost immediately after wounding and spanning several neighboring cells (indicated to the right of the kymograph).

The hybrid wound response described above was evident while embryos still displayed obvious cortical excitability. However, after the 2 cell stage, cortical excitability is slowly but progressively diminished such that in late blastulas, rather than obviously propagating waves, cortical F-actin is evident as low amplitude pulses or flickers, and in gastrula periodic F-actin behavior is essentially undetectable above the background of the apical F-actin (Swider et al., 2022). We therefore decided to assess the wound response in these later stages. In late blastulae, wounding triggered in the wounded cell an apical wave of elevated F-actin that traveled from the cell-cell junctions inward to the middle of the apical domain (Supplementary Figure S1). Occasionally, a similar wave in nearby cells was observed (Supplementary Figure S1). In early gastrulas, all of the neighbors responded to wounding by generating an increase in apical F-actin, and in later gastrulas, this response was even more pronounced (Figure 4).

The apical actin assembly spread out from the wounded cell as a rapidly (12–27 μm/s) moving wave. At the level of individual epithelial cells, the F-actin signal increased first throughout the apical domain for ∼30 s; this was followed by an increase in junctional F-actin which commenced ∼60 s after the rise in apical actin but persisted well after the apical F-actin returned to resting levels (Figures 4A–C). Measurement of the apical F-actin signal at different distances from the wound site revealed that the F-actin wave traveled away from the wound without damping (Figure 4B), suggesting that its underlying control mechanism may be based on excitable dynamics.

### Excitable calcium waves

**FIGURE 5:**
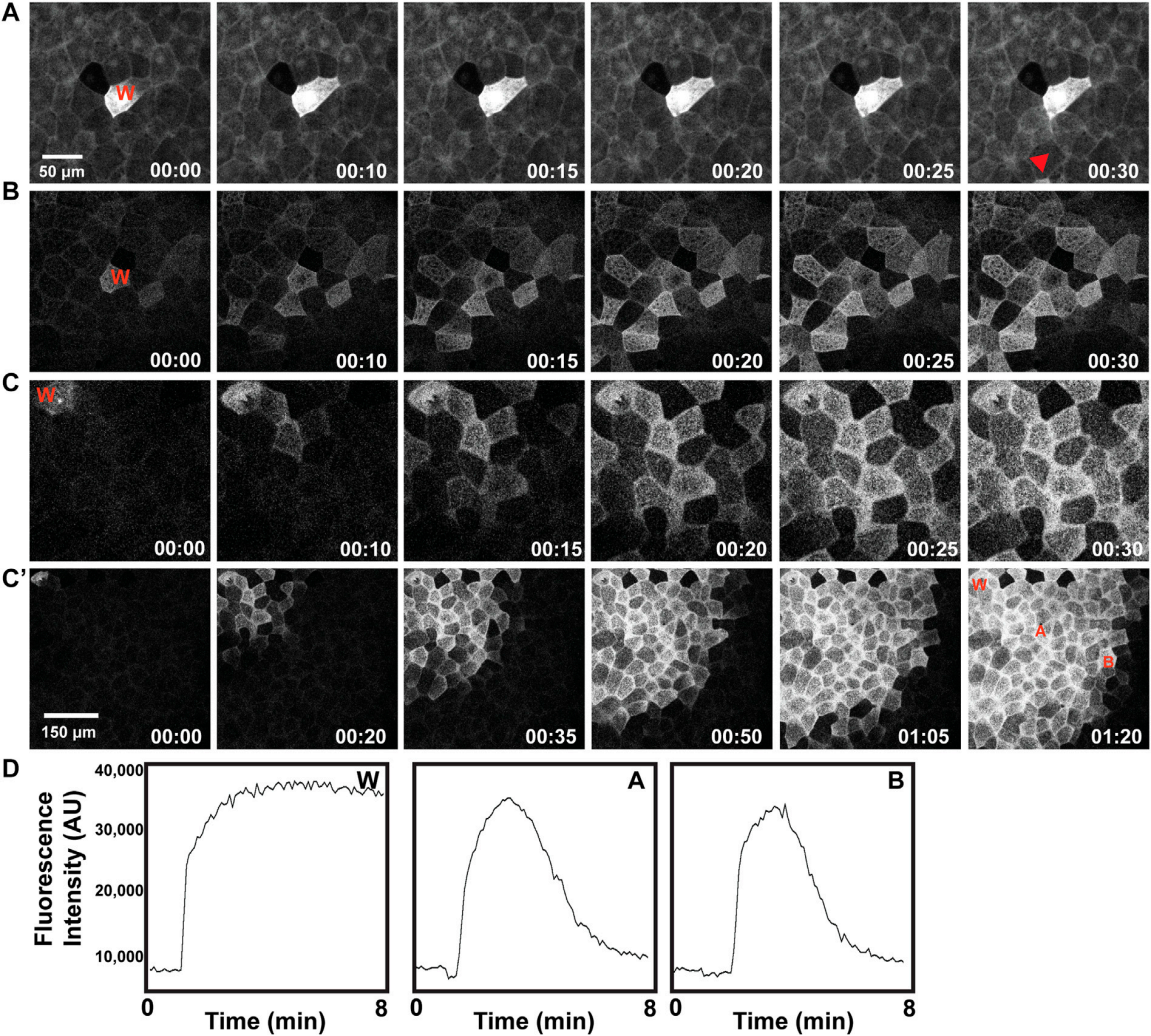
Development of a wound-induced multicellular calcium wave. Wound site indicated as “W” in each example, described as follows. **(A)** Wounded early blastula results in a localized calcium response, mainly contained within the wounded cell, shown in greyscale. **(B)** Wounded late blastula/early gastrula results in a wave of calcium extending to neighbor cells. **(C)** Wounded gastrula results in a wave of elevated calcium, extending to surrounding neighbor cells. **(C′)** Wounded gastrula, with larger surface area depicted (note scale), leading to a broadened distance of cells exhibiting calcium wave response. **(D)** Plot of fluorescence intensity (in arbitrary units, AU), of calcium over time (in minutes), as indicated via the calcium sensor GCamp6, as measured at the wound site “W”, a neighboring cell, “A”, and a distal neighboring cell, “B”, where each of these locations are identified in **(C′)**.

Wounding of *Xenopus* oocytes and early embryos triggers calcium elevation in a ring-like region bordering the wound itself and, in embryos, at the cell-cell junctions near the wound site (Clark et al., 2009; Davenport et al., 2016). Moreover, both spontaneous and wound-induced, multicellular calcium waves have been reported in various embryos and larva (Xu and Chisholm, 2011; Yoo et al., 2012; Razzell et al., 2013; Shannon et al., 2017) including *Xenopus* (Wallingford et al., 2001; Soto et al., 2013). Further, recent work from cultured cells has revealed the existence of mechanically regulated, calcium-dependent processes that link to very rapid changes in the actin cytoskeleton (Shao et al., 2015; Wales et al., 2016; Wang et al., 2019). We therefore sought to determine the relationship between calcium and wounding using the calcium sensor GCamp6 (Chen et al., 2013). Consistent with the results for the F-actin wave, wounding elicited an increase in intracellular free calcium that moved through the epithelium like a wave (Figure 5A). Further, this wave was obviously better-developed in gastrulae (Figures 5C,C') than early gastrulae (Figure 5B) or blastulae (Figure 5A), precisely paralleling the developmental characteristics of the embryonic actin wave. Further, like the actin wave, the calcium wave was also undamped (Figure 5D).

**FIGURE 6:**
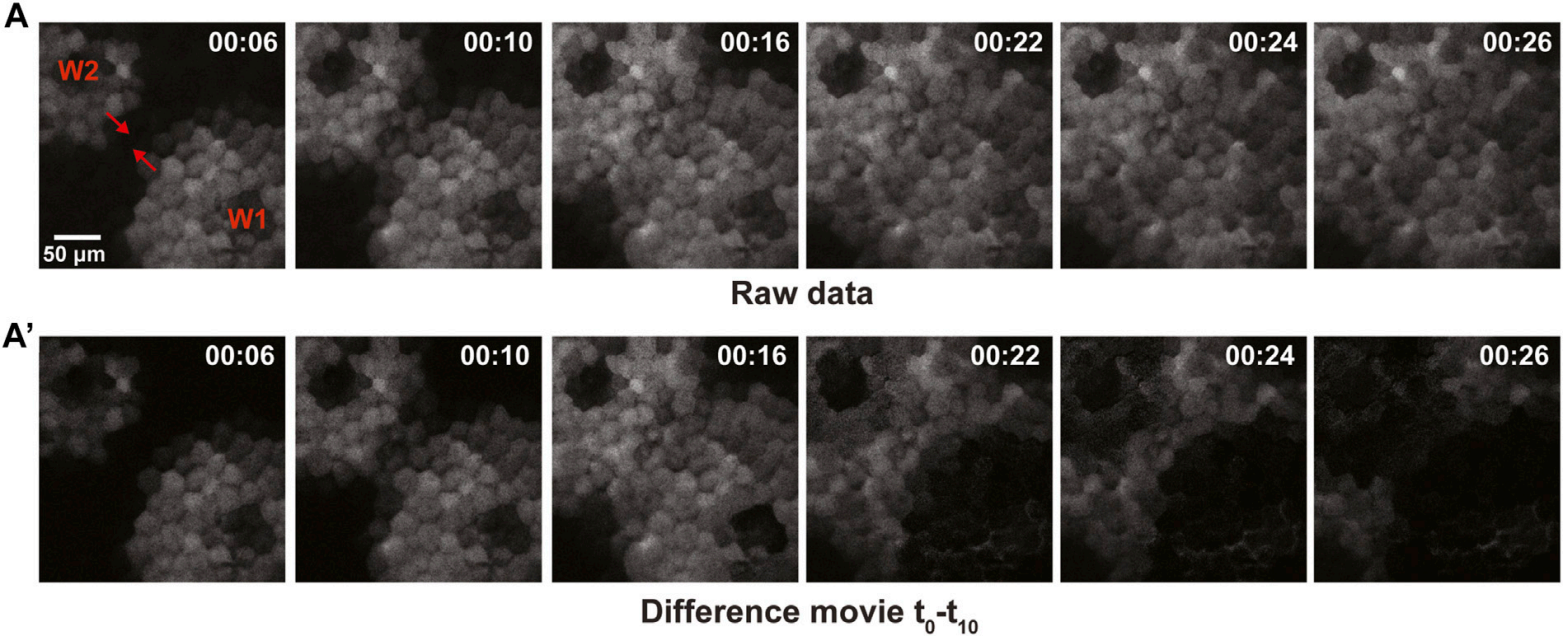
Collisions of calcium waves result in auto-annihilation in late-stage gastrula. **(A)** Raw data for imaging of two opposing wavefronts of calcium, shown in grayscale with the direction of wave propagation indicated via red arrowhead. The wavefronts approach one another, resulting in rapid auto-inhibition of the wave. **(A′)** Difference movie of the opposing wavefronts, revealing auto-inhibition of the waves shown in **(A)**.

The lack of damping is suggestive of excitable dynamics. As an alternative test of this possibility, we sought to determine if the calcium waves auto-annihilate upon collision. That is, excitable systems experience auto-annihilation as a result of the negative feedback regulating excitable wave behavior: when two waves collide, their fronts are unable to propagate through each other because they encounter the negative feedback from the opposing wave. As shown in the raw data for Figure 6, upon collision, the advancing calcium wave fronts do not obviously increase in amplitude as they pass each other, consistent with an excitable mechanism. However, the raw data do not distinguish between newly elevated calcium and calcium elevated by the wave that has just passed (Figure 6A). The raw data were therefore used to generate a difference movie (Figure 6A’; see methods) which only shows newly elevated calcium. The difference movie clearly demonstrates that the waves of newly elevated calcium annihilate each other, as expected for a mechanism based on excitable dynamics.

### Uncoupling of the calcium and F-actin waves

The foregoing results suggested two broad mechanistic possibilities concerning the F-actin and calcium waves and their relationships. First, that the calcium waves might trigger the actin waves. Second, that both mechanisms are governed by excitable dynamics. To test these hypotheses, we imaged F-actin and intracellular free calcium simultaneously in embryos using mCh-UtrCH and GCamp6, respectively. Consistent with the calcium wave triggering the F-actin wave, imaging and quantification of calcium and F-actin signal in ROIs encompassing several cells consistently revealed that the rise in calcium preceded the rise in F-actin (Supplementary Figure S2).

**FIGURE 7:**
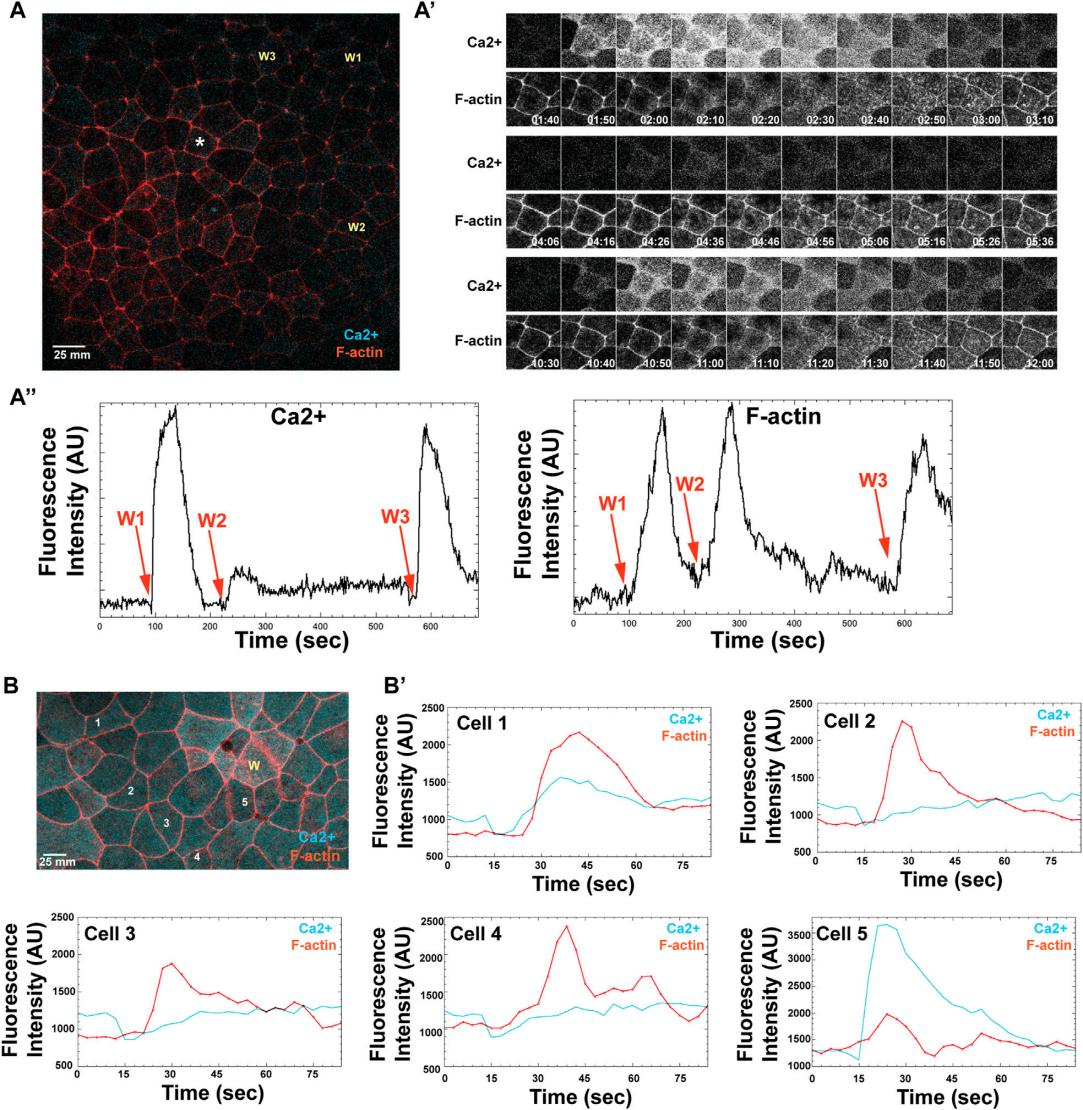
The long-distance, excitable calcium wave response in late-stage embryos is triggered independently of an apical actin response. **(A)** A gastrula stage embryo, expressing probes for both calcium (cyan) and F-actin (red), receives three successive wounds via ablation (wound locations indicated as: W1, W2, and W3). A nearby cell, marked with an asterisk, shows where calcium and F-actin responses are measured. **(A′)** Time course of calcium (top) and F-actin (bottom) response, following wounding, as measured in a single cell indicated in A (asterisk), where time is reported in minutes. W1 occurs at 1:40 min; W2 at 4:06 min; W3 at 10:30 min **(A)**″ Line scan for measured fluorescence intensity (arbitrary units, AU) for calcium and F-actin, respectively, following each wound, over time (seconds). **(B)** Late blastulae-early gastrula expressing probes for calcium and F-actin, wounded once (wound position labeled, W), with calcium and F-actin response measured in 5 cells, as indicated (labeled 1–5). **(B′)** Individual line intensity plots demonstrate the intensity of calcium and F-actin signals (arbitrary units, AU) reported over time (sec) after ablation.

An additional feature of excitable dynamics is the so-called latent period--a temporal window in which a new wave cannot be elicited because the negative feedback is still operative. To determine if the calcium and F-actin waves are subject to a latent period, wound-rewound experiments were performed. Remarkably, we found that while the calcium waves displayed a clear latent period, the F-actin waves did not. An example of one such experiment is shown in Figures 7A,A', A'': a second wound made ∼120s after the initial wound showed a very modest rise in calcium whereas one made several minutes later had a response similar to the original wound (compare arrows W1, W2, and W3 in the lefthand panel of Figure 7A). In contrast, the F-actin response following the second wound is equivalent to that in the first or the third (compare arrows W1, W2, and W3 in the righthand panel of Figure 7A). Thus, either the F-actin wave does not have a latent period or it is much shorter than the latent period for the calcium wave.

The fact that a high amplitude F-actin wave can be elicited even when the calcium wave is obviously curtailed prompted us to look more closely at the spatiotemporal relationship of F-actin and calcium in late blastula/early gastrula embryos by using ROIs that encompassed only parts of single cells rather than several cells. The results from 5 cells in one such experiment are shown in Figure 7B and B'. While in two of the cells (Cell 1 and Cell 5), the increase in F-actin clearly follows a sharp rise in calcium, in the others (Cells 2–4), the relationship is much less clear with the F-actin rise occurring at the same time or before the rise in calcium. In addition, another feature of the calcium response is evident in these experiments that we previously overlooked: wounding is actually followed by a modest but reproducible **decrease** in intracellular free calcium that occurs prior to the increase. Indeed, in cells two to four, the actin wave reaches its peak while the calcium has only returned to baseline after the decrease.

## Discussion

The first finding of this study is that wounding in zygotes and early embryos results in rapid (within seconds) freezing of nonfurrow waves and suppression of new wave formation at wound-proximal regions (i.e., areas close to wounds). Given that wave formation and propagation depend on both actin assembly and disassembly (Bement et al., 2015; Swider et al., 2022) the results imply that wounding generates a rapidly-moving signal that acts at or upstream of F-actin dynamics. Calcium is a strong possibility in that wounding elicits a rapid, ring-like increase in intracellular free calcium around wounds in frog oocytes and embryos (Clark et al., 2009; Davenport et al., 2016). What might link the calcium signal to the observed changes in cortical actin dynamics? One intriguing possibility is calcium- and calmodulin-dependent modulation of the formin, INF2, which has recently been linked to very rapid changes in the actin cytoskeleton in a variety of cultured cells (Shao et al., 2015; Wales et al., 2016; Wang et al., 2019). Of most interest is an INF2-dependent behavior referred to as CaAR (Calcium-mediated Actin Reset) which can occur as INF2 triggers actin assembly at the endoplasmic reticulum (ER) at the expense of cortical actin assembly (Wales et al., 2016). The parallels to the observed impact of wounding excitable waves are striking in that CaAR is transient, calcium-dependent, triggered by wounding (and other mechanical stimuli), and results in temporary freezing of membranous compartments. While we cannot at present say whether the freezing of the excitable actin waves in the cortex is accompanied by actin assembly associated with the ER, there is a large pool of ER in the embryos below the cortex so this notion is certainly plausible. Further, not only is INF2 expressed in *Xenopus* embryos (Wuhr et al., 2014) its characteristics make it uniquely suited to driving wave freezing in that it has the capacity to not only promote actin assembly but also severing and disassembly (Gurel et al., 2014; Gurel et al., 2015). In other words, if INF2 is a normal component of the wave-generating machinery that participates in both the assembly and disassembly of F-actin within waves, calcium-dependent recruitment of it from the cortex to some other compartment would provide a very simple explanation for the otherwise highly peculiar phenomenon of wave freezing.

Regardless of the mechanism that suppresses wave dynamics around wounds, once wound-induced flow begins, the result is a transient clearing of F-actin from the region around wounds as the frozen waves are pulled toward the wound. This behavior is similar to the clearing reported for cortical F-actin in wounded, immature frog oocytes (Mandato and Bement, 2001), with the important distinction that immature oocytes do not exhibit cortical excitability. Rather, they have a relatively inert cortical actomyosin cytoskeleton that is pulled toward wounds by virtue of the contractile ring that wounding elicits. In immature oocytes, it is apparent that this is made possible by the integration of cortical F-actin and myosin-2 (Mandato and Bement, 2001). In contrast, in embryos, the nonfurrow waves are not obviously interconnected but rather appear to operate independently of each other. Nonetheless, the current results imply strongly that they are in fact crosslinked to the rest of the cortical cytoskeleton. While such connections cannot be easily discerned in most movies, they can be seen in fixed samples stained with phalloidin to reveal details of the cortical F-actin cytoskeleton (Bement et al., 2015).

What is the functional utility of this complex series of behaviors, if any? One possibility is that healing is aided by flow-dependent increases in the amount of contractile material around the wound (Mandato and Bement, 2001; Abreu-Blanco et al., 2014). It is also plausible that the clearing promotes healing by local reduction of “competition” around the contractile array. That is, the contractile ring may close more efficiently if it need not overcome contraction in the surrounding region.

The second finding of this study is that an F-actin-rich array forms between wounds and nearby junctions and establishes what appears to be a mechanical linkage between the two. That is, previous work identified hybrid contractile rings in which junctions and wounds become unified, but the mechanism by which such structures occurred was unclear. This was at least in part because the imaging regime failed to reveal details of the wound response (Clark et al., 2009). The current results make it clear that the hybrid forms as a result of a new contractile structure which forms a contractile bridge between the two. Live imaging of the formation of this structure suggests an additional potential role of wave freezing: the frozen waves appear to become converted into–or at least contribute to–this bridge. Further, were the waves to continue in the region between the wound and the junction, it is difficult to see how the bridge could form, since wave dynamics entail constant F-actin disassembly at the wave trailing edge. To put it another way, cessation of cortical excitability between the wound and nearby junctions may be essential to establish a cortical signaling regime necessary to establish the bridges.

The signaling mechanisms that lead to bridge formation have yet to be established. Based on the potential role for CaAR discussed above, it is plausible that calcium-dependent redistribution of INF2 might contribute by way of wave freezing. In addition, recent work on junction repair in *Xenopus* embryos (Stephenson et al., 2019; Varadarajan et al., 2022), suggests the promising possibility that wound-induced changes in tension act as the upstream trigger for both calcium increases and the previously-described activation of Rho near the site of bridge formation (Clark et al., 2009). That is, during *Xenopus* development, embryos develop spontaneous breaches in their tight junctions due to tension development that arises from morphogenesis (Varadarajan et al., 2019). These breaches are repaired by local Rho activation (Stephenson et al., 2019). Rho activation is controlled by tension-dependent opening of junctional calcium channels as the junctions stretch (Varadarajan et al., 2022) and the resultant calcium **influx** somehow stimulates p115Rho GEF, thereby leading to local Rho activation and actomyosin assembly (Chumki et al., 2022). Consistent with a similar mechanism being involved here, previous work documented local calcium, Rho, F-actin, and myosin-2 increases at junctions near cell wounds (Clark et al., 2009).

Moreover, in wounds near junctions where the bridges form, a transient recoil of the junction is observed upon wounding; such recoil is not observed in wounds far from junctions where bridges fail to form (e.g., Figure 3). We therefore suggest the following series of events result in bridge formation: in wounds sufficiently close to junctions, junctional recoil triggers opening of tension-gated calcium channels at the junctions. The resultant calcium influx triggers activation of p115 RhoGEF which in turn locally activates Rho. Rho activation, combined with wave freezing, results in formation of the bridge. The bridge could then potentially be reinforced by contraction-mediated opening of additional calcium channels.

The third finding of this study is that *Xenopus* embryos develop epithelial excitability, a behavior in which wounding elicits waves of elevated calcium and increased cortical F-actin that are not merely transmitted to immediate neighbors, but instead are rapidly disseminated across the entire embryo. Strikingly, epithelial excitability develops at approximately the same developmental period that cortical excitability is being lost, at around mid-to-late blastula. This is not to say that the two processes are directly related (although see below) but rather to point out that at about the time an excitable behavior that operates at the single cell level is being lost, another that operates at a tissue level is being gained.

The evidence that multicellular waves represent excitable dynamics is strongest for calcium in that the waves are undamped, autoannihilate, and show a clear latent period. With respect to the feedbacks implicit in a wave mechanism based on excitable dynamics, we suggest that positive feedback arises from a combination of calcium-induced calcium release, possibly with IP3 as an intermediate (e.g., Soto et al., 2014). We suggest that the negative feedback is provided by a depleted substrate mechanism (Bement et al., 2024) in which the wave is based on ER stores that are depleted during wave generation (e.g., Wu et al., 2013). With respect to the F-actin wave, while it too is undamped, we have not yet tested it for autoannihilation and if it has a latent period, it must be shorter than that of the calcium wave.

How does epithelial excitability work? The mechanisms underlying epithelial excitability are intriguing and, frankly, perplexing. To begin with the features that are clear, the excitability response is distinct from the basic wound response. That is, epithelial excitability results in epithelial-wide increases in calcium, cortical F-actin, junctional F-actin, and apical F-actin that actually precede the formation of the actin ring around the wound site. Further, the basic wound response is evident well before epithelial excitability in developmental time (oocyte vs late blastula/gastrula). Likewise, the “bridge” response appears in development much sooner than epithelial excitability (32-cell embryo vs. late blastula/gastrula).

We are also confident that the calcium and F-actin waves that comprise epithelial excitability arise from a common upstream signal. This assertion is based on the fact that they both appear at mid/late blastula become more pronounced over the same developmental window--mid/late blastula to gastrula. Moreover, the calcium and F-actin waves propagate at similar speeds, both between embryos and when compared directly in the same embryos (e.g., Supplementary Figure S2).

However, we remain puzzled about other mechanistic features of embryonic excitability. For example, we initially assumed that the calcium wave triggered the F-actin wave. However, this conclusion is severely undermined by the wound-rewound experiments and the single cell analysis of waves in late blastulae, which indicate that the cortical F-actin can increase with minimal or even no increase in calcium. Moreover, high temporal imaging of changes in intracellular free calcium indicates clearly that the most consistent feature of the calcium response following wounding in both late blastulae and gastrulae is actually a small *decrease* in calcium. This decrease is either followed by a return to resting levels or a rapid rise to levels characteristic of the wave. However, because F-actin can rise under both of these conditions, it is difficult to escape the conclusion that a decrease in calcium may actually serve as the signal upstream of the F-actin wave. While we do not wish to overburden CaAR and INF2 as potential explanations for the results presented here, it seems at least possible that the drop in calcium might essentially trigger the opposite of CaAR (Wales et al., 2016): an increase in cortical F-actin at the expense of an ER-related pool. Such a scenario prompts two obvious questions, namely, what is the upstream signal responsible for the calcium drop? And why are its effects not reversed by the subsequent calcium rise? Answering such questions will require further study.

Finally, what is the point of epithelial excitability? Obviously, a mechanism for long-range communication could be of great use during morphogenesis to help coordinate tissue movements. But we are also struck by the fact that this rapid system of communication is coupled to mechanics via the apical actin waves. Perhaps epithelial excitability represents a means for the embryo to generate coordinated, tissue-level mechanical changes in respond to extracellular stimuli—like wounds—prior to the development of the muscle and nervous system (Kim et al., 2014).

## Methods

### Embryo preparation

Mature *Xenopus laevis* females were injected in the dorsal lymph sac with 800 U of human chorionic gonadotropin (MP Biomedicals) and incubated at 18°C for 12–18 h before use to induce ovulation. Eggs were laid into a 1 x high-salt Modified Barth’s Saline (high salt MBS; 108 mM NaCL, 0.7 mM CaCl_2_; 1 mM KCL; 1 mM MgSO_4_; 5 mM HEPES; 2.5 mM NaHCO_3_). Eggs were fertilized *in vitro* using surgically acquired, homogenized testes tissue from *Xenopus laevis* males; 15–30 min following fertilization, embryos were dejellied in a solution of 2% cysteine in a 0.1 x Marc’s Modified Ringers’ (MMR; 100 mM NaCl, 2 mM KCl; 1 mM MgCl2; 5 mM HEPES; pH 7.4) and rinsed thoroughly with 0.1 x MMR. Embryos were injected once at the one-cell stage (30–45 min following fertilization) in 5% Ficoll (Sigma) in 0.1 x MMR with 5 nL of *in vitro* synthesized mRNA and maintained in 0.1 x MMR during development.

### Constructs, mRNA synthesis, and microinjection

Plasmids for eGFP-UtrCH, mCherry-UtrCH (Burkel et al., 2007), and GCamp6 (Chen et al., 2013) were made as described previously. Plasmids were linearized following the open reading frame and used as a template for mRNA synthesis and using the mMessage Machine SP6 kit (Ambion). mRNAs were polyadenylated using a Poly(A) tailing kit (Ambion) and purified with a phenol-chloroform extraction and isopropanol precipitation. mRNA constructs were run on an RNA gel to assess quality, then microinjected into embryos, as necessary.

### Microscopy and wounding

Embryos were maintained in 0.1 x MMR at 18°C and imaged at room temperature (20–24°C). For confocal experiments, embryos were gently compressed with a clean #1.5 coverslip and sealed in rings of vacuum grease.

Frog embryos were collected on a Prairie View Laser Scanning Confocal on a Nikon Eclipse Ti inverted microscope base (Bruker Nano Surfaces), and imaging was conducted through Prairie View software. Data were collected via using a 40x/1.0 NA objective or a ×60/1.4 NA oil immersion objective.

Wounding was performed via ablation, as previously described (Benink and Bement, 2005). Briefly, a laser at a pulse of 440 nm was fired into the sample from a nitrogen pump laser (Laser Sciences). Wounds were made in the animal hemisphere of embryos.

### Image processing

Image processing was conducted using ImageJ/FIJI. Kymographs were generated in FIJI by reslicing time-lapsed image data along a 1 pixel-wide line drawn across the field of view at the locations indicated on individual figures and using bicubic interpolation to stretch the *y*-axes for display. Figures were assembled in Adobe Illustrator. Difference movies were generated in FIJI by duplicating the original movie file, removing 10 frames from the beginning of one copy, 10 frames from the end of the other copy, and then subtracting the second movie copy from the first.
