# Supplementary figures and images for: Bring the pain: wounding reveals a transition from cortical excitability to epithelial excitability in Xenopus embryos
Source: Front Cell Dev Biol. 2024 Feb 22;11:1295569. doi: 10.3389/fcell.2023.1295569 (PMC10918254; doi:10.3389/fcell.2023.1295569)

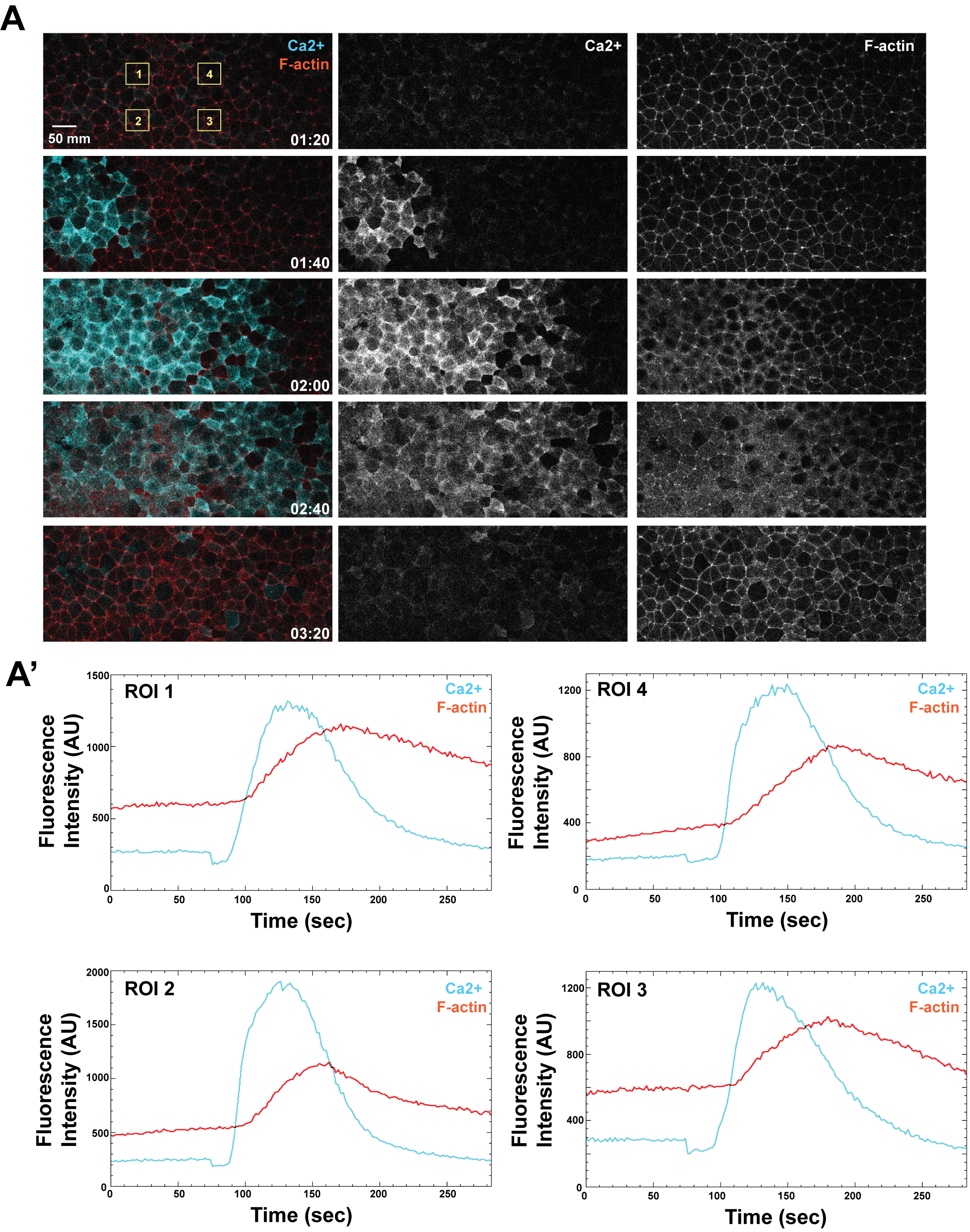

Supplement: Supplementary file 3 [file Image2.tif]

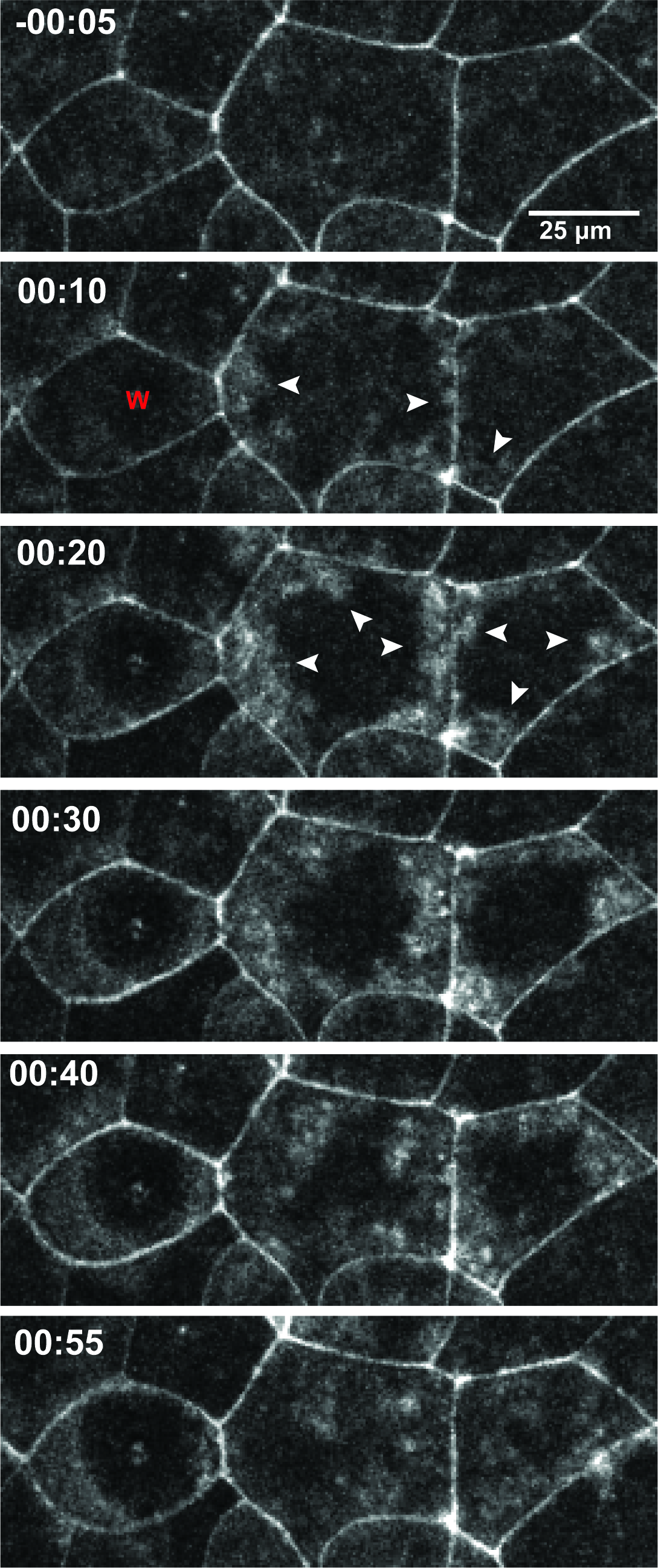

Supplement: Supplementary file 4 [file Image1.tif]
