# Supplementary material for: Bring the pain: wounding reveals a transition from cortical excitability to epithelial excitability in Xenopus embryos
Source: Front Cell Dev Biol. 2024 Feb 22;11:1295569. doi: 10.3389/fcell.2023.1295569 (PMC10918254; doi:10.3389/fcell.2023.1295569)
Supplement: Supplementary file 5 [file DataSheet1.pdf]

**Supplementary Material**

**Supplementary Figure 1: Wound-induced waves of F-actin originating from the junctions and spreading inward.** Representative images of a late blastula, with wound ("W"). Neighboring cells show apical waves of F-actin traveling from the junctions (elevated regions of F-actin indicated via white arrowheads); these waves of F-actin then travel inward, across the apical surface of the cell (arrowheads indicate inward progression). Timing relative to the time of wounding.

**Supplementary Figure 2: F-actin wave consistently lags behind the calcium wave.**
(A) Representative images of a mid/late blastula co-expressing calcium and F-actin probes, with four representative regions of interest (yellow, boxed ROI labeled 1-4); regions encompass more than one cell per region. Timing relative to the time of wounding. (A') Line traces for each of the four regions of interest, as labeled in A, with responding calcium and actin intensity (arbitrary units, AU) against time (seconds) after wounding (wound location not shown).

**Movie Legends**

**Movie 1: Cortical F-actin waves as observed in *Xenopus* embryo. Corresponds to Figure 1 (movie width corresponds to 600 μm).**

**Movie 2: Wounding results in contractile ring formation, F-actin wave freezing, pulling of frozen waves toward the wound, and absence of new, wound-proximal wave formation. Corresponds to Figure 2 (movie width is 75 μm).**

**Movie 3: A junction-proximal wound pulls upon the nearby junction, whereas a junction-distal wound does not. Corresponds to Figure 3 (movie width is 250 μm).**

**Movie 4: Wounding of gastrula results in an apical increase of F-actin; this F-actin response spreads to neighboring cells. Corresponds to Figure 4 (movie width is 200 μm).**

**Movie 5: Wounding of late gastrula results in a propagating calcium wave, which spreads beyond immediately neighboring cells. Corresponds to Figure 5 (movie width is 500 μm).**

**Movie 6: Difference movie showing that calcium waves auto-annihilate. Corresponds to Figure 6 (movie width is 250 μm).**
